# Paclitaxel induced-mechanical hypersensitivity is associated with time-dependant pro-inflammatory galectin-3^+^ macrophages accumulation in the dorsal root ganglia

**DOI:** 10.1016/j.ynpai.2026.100219

**Published:** 2026-05-25

**Authors:** Lynda Zeboudj, Marzia Malcangio

**Affiliations:** Wolfson, Sensory, Pain and Regenerative Centre, King's College London, London SE1 1UL, UK

**Keywords:** Macrophages, Paclitaxel-induced neuropathic pain, Pro-inflammatory macrophages, Galectin-3, Chronic pain

## Abstract

Neuropathic pain is a dose-limiting side effect of chemotherapeutic agents such as paclitaxel, and it is unsuccessfully treated by available analgesics. Among the mechanisms underlying paclitaxel-induced neuropathic pain, neuroimmune interactions in the dorsal root ganglia (DRGs) play a pronociceptive role. However, how immune responses evolve over time during pain maintenance and persistence is poorly understood.

Here we treated mice with six injections of paclitaxel (PTX) every other day over 10 days (cumulative dose of 15 mg/kg) and detected mechanical hypersensitivity that developed at day 7 and lasted up to day 28 after first PTX injection. We observed a time-dependent accumulation of macrophages in lumbar DRGs at day 28, but not day 14 after first PTX injection. At day 28, proinflammatory macrophages MHCII^+^CD206^−^ accumulate in DRG together with the emergence of galectin-3 expressing macrophages. Galectin-3 expression was also found in splenic monocytes mirroring the DRG macrophage phenotype and pointing to a coordinated, compartment specific remodelling of innate immune responses. Early adaptive immune responses were spatially and temporally restricted with transient increase in CD8^+^ T cells in the DRG at day 7 while splenic regulatory T cells were selectively reduced 28 days after first PTX injection.

Together these findings reveal that PTX treatment drives coordinated immune responses, and emergence of galectin-3 pro-inflammatory macrophages in DRG could be a feature of persistent paclitaxel-induced neuropathic pain.

## Highlights

• DRG proinflammatory macrophage numbers peak with persistent neuropathic hypersensitivity.
• Galectin-3-expressing macrophages emerge in DRG in concomitance to persistent pain.
• Adaptive immune responses are temporally and spatially organised in response to paclitaxel treatment.

## 1 Introduction

Chemotherapy-induced peripheral neuropathy (CIPN) represents one of the most adverse effects of cancer treatment and it is associated with the use of chemotherapeutic agents such as taxanes, platinum based compounds and vinca alkaloids (Grisold et al., 2012; Seretny et al., 2014). CIPN is mainly characterised by sensory disturbance including numbness, burning, tingling, mechanical allodynia and ongoing pain. Such neuropathic pain symptoms may persist long after treatment completion, significantly affecting patients' quality of life and cancer therapy adhesion. Moreover, CIPN may require dose reduction or termination potentially compromising treatment efficacy (Burgess et al., 2021; Staff et al., 2017). Therefore, there is a critical need to better understand the underlying mechanisms for pain in CIPN and identify innovative analgesic strategies.

Paclitaxel, a widely used taxane chemotherapeutic agent, is among the most common agent associated with peripheral neuropathy. It is mainly used for solid tumours such as ovarian, breast and non-small cell lung carcinoma (Jaggi and Singh, 2012). While it stabilises microtubules to prevent cancer cell division paclitaxel also disrupts axonal transport and neuronal transport in peripheral sensory neurons. Indeed, paclitaxel can lead to mitochondrial dysfunction, oxidative stress and axonal degeneration, which all contribute to the development of neuropathic pain (Klein and Lehmann, 2021). In addition to direct neuronal effects, increasing evidence highlights a key role for neuroimmune interactions in the development of paclitaxel-induced neuropathy. Indeed, macrophages accumulate in peripheral nerves and dorsal root ganglia (DRG) following paclitaxel treatment and promote nociceptor sensitization and the development of persistent pain through the release of pro-inflammatory mediators such as TNF-α, IL-6, IL-1β and suppression of anti-inflammatory mediator release such as IL-10 (Lees et al., 2017; Peters et al., 2007). However, while macrophage involvement in chronic pain mechanisms is well established, the temporal dynamics of their recruitment and activation in DRG, remain incompletely understood.

Beyond innate immunity, emerging evidence suggests that adaptive immune responses may also contribute to CIPN. Indeed, experimental studies have reported modest changes in DRG T-cell population in concomitance to paclitaxel induced peripheral neuropathy, including infiltration of CD4^+^ T helper cells and CD8^+^ cytotoxic T cells (Krukowski et al., 2016; Laumet et al., 2019). However, the spatial and temporal organisation of adaptive immune responses across neural and peripheral immune compartments is still poorly defined. Importantly, it is not known whether paclitaxel induces a global systemic immune response or instead drives compartment-specific immune remodelling. Furthermore, how innate and adaptive immune responses change over time to sustain neuroinflammation remains to be determined.

Galectin-3 (Gal-3), a β-galactoside-binding lectin highly expressed by activated macrophages, has emerged as an important regulator of immune responses and neuroinflammation. Previous studies have shown that Gal-3 modulates microglial and macrophage activation in the nervous system and contributes to inflammatory signalling pathways involved in pain mechanisms. For instance, Gal-3 released from nociceptive afferents activates toll-like receptor 4 (TLR-4)-dependant microglial responses in the spinal cord and promotes inflammatory allodynia (Sideris-Lampretsas et al., 2023). Furthermore, paclitaxel treatment increases Gal-3 expression in Schwann cell in peripheral nerves, and Gal-3 application to the peripheral nerves induces monocyte-macrophage infiltration and mechanical hypersensitivity in naïve mice (Koyanagi et al., 2021). Despite these observations, whether expression of Gal-3 follows the temporal sequence of neuroimmune responses in CIPN remains unclear.

In this study, we investigated the temporal and spatial dynamics of immune responses in a mouse model of paclitaxel-induced neuropathy with a particular focus on differences in innate and adaptive immune cell populations in the DRG and across systemic immune compartments, including the spleen, blood, and lymph nodes. Given the known role of macrophages in neuroinflammation and the emerging involvement of Gal-3 in inflammatory signalling, we further examined whether Gal-3 expression is altered in myeloid cell populations during paclitaxel-induced neuropathy.

## 2 Material and methods

### 2.1 Animals

C57BL/6 mice (Charles River) were used, including both males and females aged 8–12 weeks. Animals were housed in Biological Service Unit at King's College London, under standard conditions in a temperature-controlled environment on a 12 h light/12 h dark cycle, with ad libitum access to food and water. Mice were randomly assigned to experimental groups (vehicle or paclitaxel-treated), with age-matched animals used across conditions. Where indicated, both sexes were initially included; however, as no significant sex differences were observed in behavioural responses, subsequent analyses focused on male mice. All procedures were carried out under personal and project licences granted by the UK-Home Office (project licence PP4092047).

### 2.2 Paclitaxel treatment

**Fig. 1:**
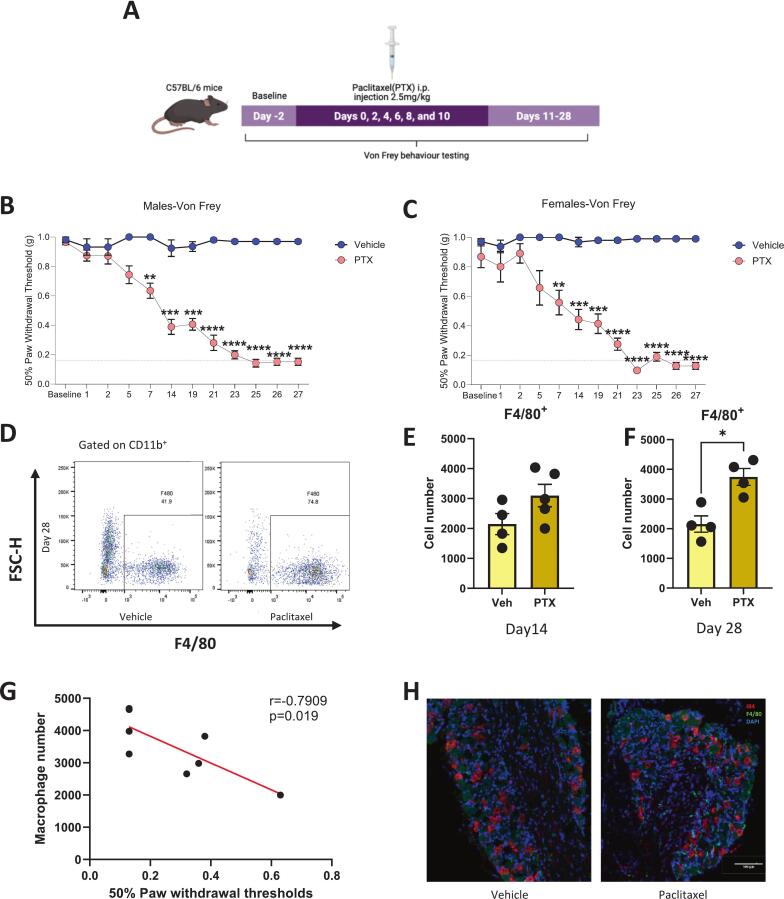
Paclitaxel induces mechanical hypersensitivity and accumulation of macrophages. A Schematic of the experimental protocol showing paclitaxel (PTX) administration and behavioural testing. *n* = 4–8 males and n = 4–8 females, 2 groups (Vehicle control, and Paclitaxel at 2.5 mg/kg for a cumulative dose of 15 mg/kg). B Attenuated mechanical hypersensitivity in paclitaxel group in males and **C** in females up to day 27 in paclitaxel treated group. Data are presented as 50% paw withdrawal thresholds (PWT). ***P < 0.001 compared with vehicle thresholds; by 2-way ANOVA followed by Tukey's multiple-comparison test (*n* = 4–8 per group). D–F Flow cytometric analysis of dorsal root ganglia (T10–L5) showing increased CD11b^+^F4/80^+^ macrophage numbers in paclitaxel-treated at day 28 but not at day 14, *n* = 4. G Person's correlation between macrophage number and paw withdrawal threshold, *n* = 7. H Representative immunofluorescence of F4/80 (green), IB4 (red) and DAPI (blue) in DRG vehicle and paclitaxel at day 28, Scale bar =100um. Data are presented as mean ± SEM. *P < 0.05; by unpaired, 2-tailed Student's *t-*test. (For interpretation of the references to colour in this figure legend, the reader is referred to the web version of this article.)

Mice received paclitaxel (Intaxel, Fresenius KABI limited) or vehicle (20% Cremophor EL/saline) intra-peritoneally at a dose of 2.5 mg/kg every other day for a total dose of 15 mg/kg (days 0, 2, 4, 6, 8, and 10) (Fig. 1A). This protocol was validated by pilot experiments to produce robust, and reproducible mechanical hypersensitivity as reported by others (Sankaranarayanan et al., 2023). Mice were randomly assigned to experimental groups and weighed prior to each injection. Injection volumes were adjusted according to body weight to ensure accurate dosing.

### 2.3 Behavioural testing

Mechanical hypersensitivity was assessed using calibrated series of von Frey filaments (0.02-1 g) applied to the plantar surface of the hind paw. Mice were placed individually in testing compartments and allowed to habituate for 30 min to 1 h before of the application of the 0.07 g filament, which was applied until a withdrawal response was elicited. Mechanical thresholds were determined using the up–down method, where a positive response to the 0.07 g filament resulted in the application of a filament with lower force, whereas a no response was followed by application of a higher force filament. This process continued until a change in response was observed or until the highest filament (1 g) failed to elicit a response (Chaplan et al., 1994). The 50% paw withdrawal threshold was calculated using Dixon's method (Dixon, 1980).

### 2.4 Flow cytometry

Mice were deeply anesthetized by intraperitoneal injection of pentobarbital (Pentoject). Whole blood was collected via intracardiac puncture, and this was followed by transcardial perfusion of ice-cold PBS. Dorsal root ganglia (DRG), spleens, and inguinal lymph nodes were rapidly dissected and transferred into ice-cold HBSS (Gibco). Based on the assumption that paclitaxel reaches all DRGs (Livni et al., 2022; Peters et al., 2007), L3-L5 DRGs were isolated because neurons innervate the hind limb, and T10 to T13 were used to increase cell yield for flow cytometry. DRGs were enzymatically dissociated to generate single-cell suspensions using a digestion solution containing 3 mg/mL dispase (Roche), 0.125% collagenase (Sigma-Aldrich), and 200 U/mL DNase I (Sigma-Aldrich) prepared in F-12 medium (Gibco). Samples were then incubated at 37 °C for 30 min, followed by centrifugation at 300 ×*g* for 10 min. Cell pellets were resuspended in FACS buffer. Spleens and inguinal lymph nodes were mechanically dissociated by gentle disruption and passed through a 70 μm cell strainer to generate single-cell suspensions. Cell viability was assessed using Live/Dead Fixable Near-IR dye (Invitrogen, L10119) for 30 min prior to staining with fluorochrome-conjugated antibodies. Single cell suspensions were stained with anti-CD16/32, VioBlue-conjugated anti-CD11b, APC-conjugated anti-F4/80, PerCP-Cy5.5-conjugated anti-MHCII, FITC-conjugated anti-CD206, PE-Cy7-conjugated anti-Galectin-3, PerCP-Cy5.5-conjugated anti-Gr-1, and BV605-conjugated anti-CD115 were used for meyloid cell characterisation and phenotyping. BV421-conjugated anti-CD4, BV711-conjugated anti-CD4, BV510-conjugated anti-CD8, FITC-conjugated anti-CD8, PerCP-Cy5.5-conjugated anti-CD8, APC-conjugated anti-CD3, PE-conjugated anti-CD62L, BV421-conjugated anti-CD44, FITC-conjugated anti-CD25, BV421-conjugated anti-FoxP3, PE-conjugated anti–IL-17, and BV605-conjugated anti–IFN-γ were used for T cell characterisation. All antibodies were obtained from BioLegend and used at a dilution of 1:200. Cells were analysed using a flow cytometer (BD LSRFortessa).

### 2.5 Immunofluorescence

For immunofluorescence analysis, mice were transcardially perfused with ice-cold PBS, and DRG (L3-L5) were rapidly collected and fixed in 4% paraformaldehyde (PFA; Sigma-Aldrich), then transferred to PBS-sucrose 30%. DRGs were cryosectioned at 10 μm thickness using a cryostat (Bright Instruments) and mounted on Superfrost Plus microscope slides (Thermofisher). Sections were permeabilized in PBS containing 0.1% Triton X-100, and non-specific binding sites were blocked using PBS containing 5% bovine serum albumin (BSA) for 1 h, then incubated overnight with antibodies against IBA1 antibody (1:1000, Wako chemicals), and IB4 (11,000; thermofisher scientific) followed by appropriate fluorophore-conjugated secondary antibodies (Alexa Fluor488, Alexa Fluor546; 1:1000; Invitrogen). Fluorescent images were acquired using a Zeiss LSM710 confocal microscope and processed using ZEN software (Zeiss).

### 2.6 Statistics

All data are expressed as mean ± SEM. GraphPad Prism was used to perform data analyses by unpaired Student's *t-*test (2 groups), or 2-way ANOVA followed by Tukey's multiple-comparison test (behavioural data). *P* values of less than 0.05 were considered as significant.

## 3 Results

### 3.1 Paclitaxel induces persistent mechanical hypersensitivity and accumulation of macrophages in the dorsal root ganglia

To examine immune cell changes associated with paclitaxel-induced mechanical allodynia, we treated C57BL/6 mice with paclitaxel (2.5 mg/kg, i.p. every other day from day 0 to day 10 for a total of *6* injections) or vehicle (Fig. 1A). As expected (Livni et al., 2022; Sankaranarayanan et al., 2023) paclitaxel-treated male and female mice developed a significant reduction in mechanical withdrawal thresholds from day 7 (after 4 injections) compared with vehicle-treated mice (Fig. 1B and C). This hypersensitivity exacerbated after the last injection between day 14 and day 23 and persisted until day 27. Since pain-like behaviour was not sex-dependent, we continued our studies in male mice and began with the examination of macrophage accumulation in DRG.

Flow cytometry analysis of F4/80^+^ cells (macrophages) in dorsal root ganglia (DRG) (T10–L5) revealed a trend for higher macrophage numbers relative to control at day 14 that was 4 days after the last paclitaxel injection. Then, at day 28, macrophage number in the paclitaxel DRG became significantly higher than in control DRG at 2 weeks after the last paclitaxel injection (Fig. 1D–F). The temporal accumulation of DRG macrophages correlated with mechanical hypersensitivity as shown by Pearson's correlation (*r* = −0.7909, *p* = 0.0194) (Fig. 1G). Furthermore, DRG macrophage accumulation was confirmed by immunofluorescence staining at day 28 (Fig. 1H). These findings suggest that macrophage recruitment to DRG represent a gradual neuroimmune response to paclitaxel treatment and support a link between sustained innate immune recruitment within the DRG and CIPN associated chronic pain.

### 3.2 Paclitaxel induces accumulation of pro-nociceptive macrophages and increases galectin-3 expressing macrophages in the dorsal root ganglia at chronic stages of CIPN

To determine whether macrophage phenotype evolves over time following paclitaxel treatment, we assessed the expression of canonical activation markers, MHCII and CD206, associated with pro- and anti-inflammatory macrophages, respectively (Nahrendorf and Swirski, 2016).

**Fig. 2:**
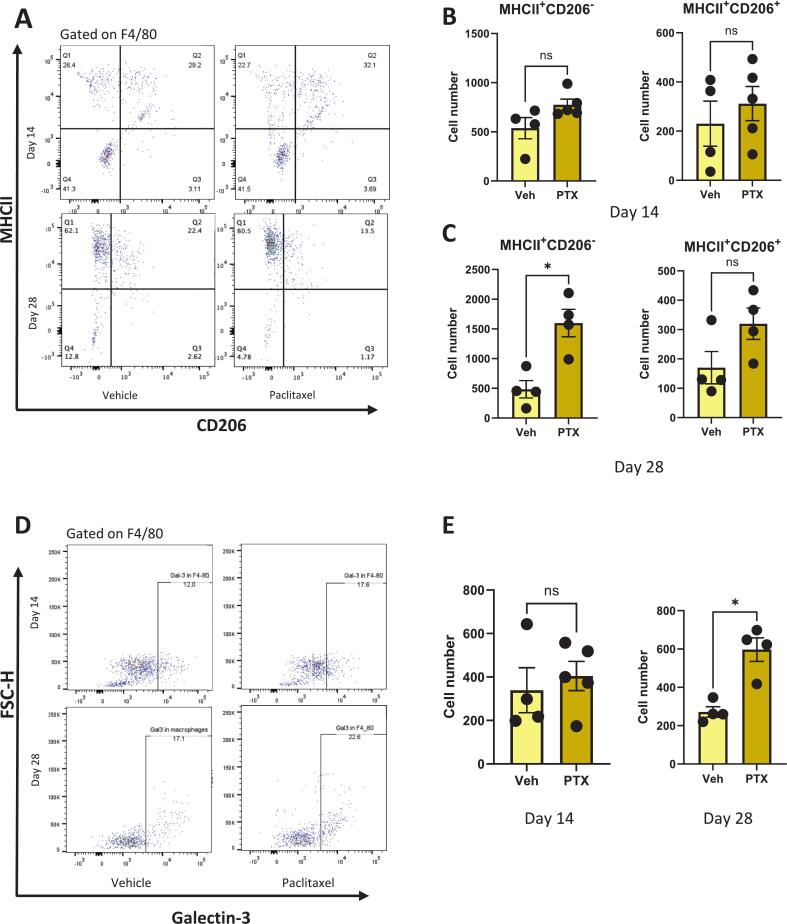
Paclitaxel-treatment is associated with accumulation of pro-inflammatory and galectin-3 -expressing macrophages in DRG. A representative scatterplot of MHCII and CD206 markers expression on CD11b^+^F4/80^+^ in DRG from day 14 and day 28 after paclitaxel compared with vehicle. B Bar graphs represent MHCII^+^ CD206^−^ (pro-inflammatory) macrophages (left panel), and the right panel represents MHCII^+^ CD206^+^ transitioning macrophages, n = 4. C increase of MHCII^+^ CD206^−^ macrophage numbers (left panel) and no difference in transitioning macrophage numbers MHCII^+^ CD206^+^ (right panel), n = 4. D Representative scatterplot of Gal −3 expressing macrophages. E no change in Gal-3-expressing macrophage numbers at day 14 (left panel) and increase of Gal-3-expressing macrophages at day 28 (right panel), n = 4. Data are presented as mean ± SEM. *P < 0.05; by unpaired, 2-tailed Student's *t-*test.

At day 14, we observed no difference in pro-inflammatory macrophages (MHCII^+^CD206^−^) and no difference in numbers of transitioning macrophages (MHCII^+^CD206^+^) in paclitaxel-treated compared with vehicle-treated DRGs (Fig. 2B). At day 28, we observed a significant increase in pro-inflammatory macrophages (Fig. 2C, left panel), and no difference in transitioning macrophages (Fig. 2C, right panel). These results support the proposal that pro-inflammatory macrophages contribute to paclitaxel-induced hypersensitivity as reported before (Peters et al., 2007).

Gal-3 has emerged as an important regulator of neuroinflammation. For instance, microglia-derived Gal-3 can function as an endogenous ligand for TLR-4, promoting inflammatory signalling (Burguillos et al., 2015). Additionally, Gal-3 is elevated in Schwann cells following paclitaxel treatment, acting as a chemoattractant for monocytes-macrophages (Imai et al., 2017; Koyanagi et al., 2021). We therefore investigated the expression of Gal-3 in DRG-macrophages, and peripheral immune compartments. At day 14, Gal-3 expression in DRG macrophages was not altered following paclitaxel treatment (Fig. 2E), indicating that early macrophage accumulation and phenotypic skewing occur independently of Gal-3 upregulation. In contrast, at day 28, we found a significant increase in Gal-3-expressing macrophages after paclitaxel treatment (Fig. 2E right panel), suggesting engagement of Gal-3-dependent neuroimmune signalling pathways at chronic stages of CIPN.

### 3.3 Paclitaxel induces compartment-specific remodelling of innate immune populations in the spleen but not immune cells in circulation

To determine whether paclitaxel-induced neuroimmune changes extend beyond the DRG, and the contribution of the systemic innate immune system to the changes occurring in the latter, we sought to analyse innate immune cell populations in the spleen and peripheral blood.

**Fig. 3:**
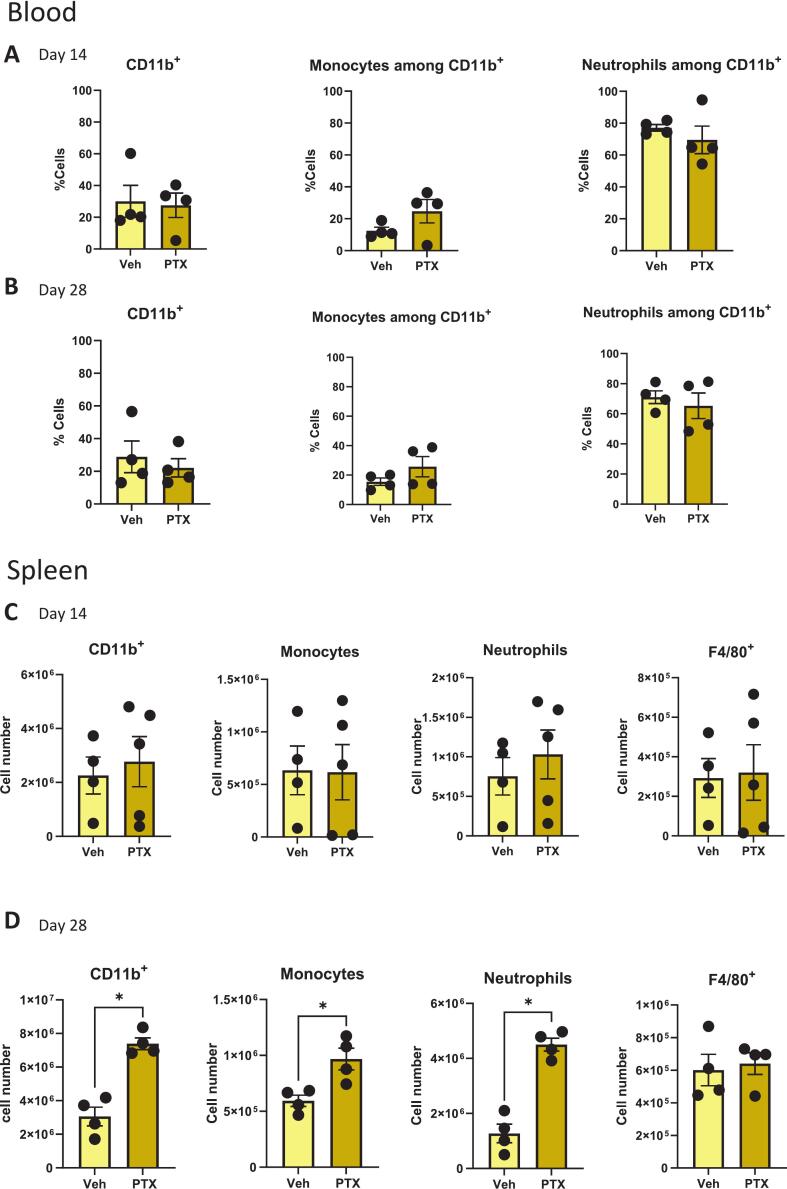
Paclitaxel treatment alters innate immune populations in the spleen but not in blood. A Bar graphs representing CD11b^+^, monocytes and neutrophils in blood of mice treated with paclitaxel compared to vehicle group at day 14 and B at day 28, n = 4–5. C No changes in CD11b+, monocytes, neutrophils and F4/80^+^ in spleen after paclitaxel treatment at day 14, n = 4–5. D In contrast, at day 28 paclitaxel-treated mice exhibited increased myeloid populations, driven by an increase in monocytes, and neutrophils with no difference in F4/80^+^ macrophages, n = 4–5. Data are presented as mean ± SEM. *P < 0.05; by unpaired, 2-tailed Student's *t-*test.

At day 14 following paclitaxel treatment, we observed no significant differences in total CD11b^+^ myeloid cells, including monocytes, neutrophils, and F4/80^+^ macrophages, in either spleen or blood (Fig. 3A, C). This indicates that early systemic innate immune compartments remain largely unaffected, despite the onset of neuropathic pain-like behaviour. In contrast, at day 28 after paclitaxel treatment, we found significant alterations in splenic innate immune populations (Fig. 3D). These changes were characterised by a significant increase in the CD11b^+^ cells, including an increase in monocytes, and neutrophils, and no difference in F4/80^+^ macrophages, consistent with a reorganisation of splenic myeloid cell composition at chronic stages. Notably, these alterations were not mirrored in the peripheral blood (Fig. 3B), where circulating CD11b^+^ cells, including monocytes and neutrophils, remained unchanged between paclitaxel- and vehicle-treated samples. Together, these findings indicate that paclitaxel induces compartment-specific remodelling of the myeloid cells, with delayed and selective changes in splenic CD11b^+^ populations rather than a global expansion of circulating innate immune cells.

### 3.4 The late upregulation of galectin-3 in splenic CD11b^+^ myeloid cells mirrors DRG macrophage activation

**Fig. 4:**
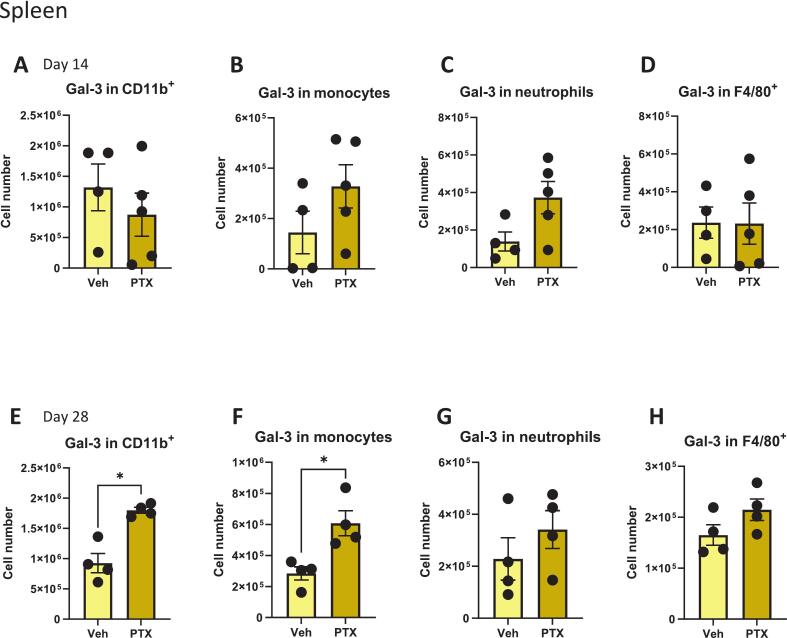
Paclitaxel treatment results in increase of galectin-3-expressing splenic CD11b^+^ cells at day 28. A–D Gal-3 expression in splenic CD11b^+^ cells at day 14, n = 4–5. E–H upregulation of Gal-3 expressing splenic CD11b^+^ subsets at day 28, which is driven by an upregulation of Gal-3 expressing monocytes (F), while no difference in Gal-3 expressing neutrophils (G) and f4/80 macrophages (H), n = 4. Data are presented as mean ± SEM. *P < 0.05; by unpaired, 2-tailed Student's *t-*test.

Given the delayed upregulation of Gal-3 observed in DRG macrophages, we next assessed its expression within peripheral myeloid cells, focusing on splenic CD11b^+^ cell subsets. At day 14 following paclitaxel treatment, Gal-3 expression within the splenic CD11b^+^ cells including monocytes, neutrophils, and F4/80^+^ macrophages was not significantly altered (Fig. 4A–D), consistent with the absence of early systemic myeloid remodelling.

However, at day 28 after paclitaxel treatment we found a significant increase in Gal-3 expression within CD11b^+^ cells (Fig. 4E), which was driven primarily by an increase in Gal-3 expressing monocytes (Fig. 4F), as we observed changes in neither neutrophils nor macrophages expressing Gal-3 (Fig. 4G and H). This temporal pattern mirrors the changes observed in DRG macrophages and supports the existence of a coordinated, time-dependent activation profile across myeloid compartments. Taken together, these findings indicate that Gal-3 upregulation is a feature of chronic myeloid activation, rather than an early response to paclitaxel.

### 3.5 Paclitaxel drives a reduction of regulatory T cells paralleling chronic myeloid cells remodelling

**Fig. 5:**
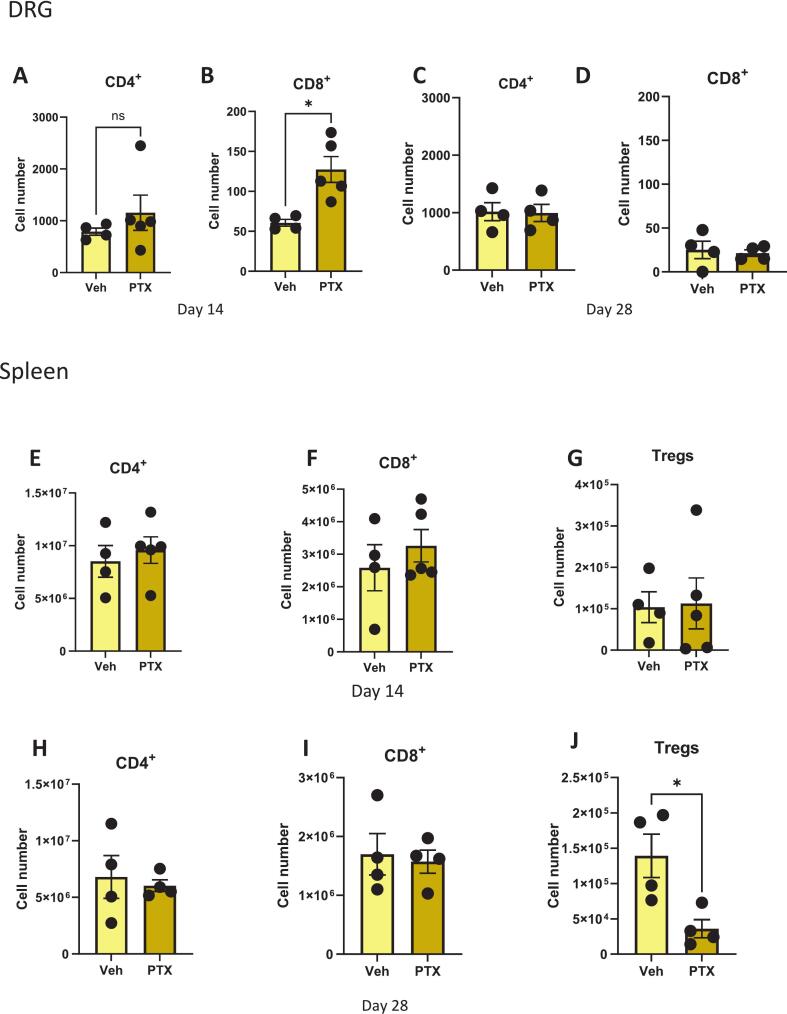
Paclitaxel treatment increases DRG CD8+ T cells at day 14 and reduces splenic regulatory T cells at day 28. (A, B) CD4^+^ and CD8^+^ T cells in the DRG at day 14 and (C, D) at day 28, n = 4–5. (E–G) Splenic T cell subsets including CD4^+^, CD8^+^ and regulatory T cells respectively at day 14, and (H–I) at day 28. n = 4–5. Data are presented as mean ± SEM. *P < 0.05; by unpaired, 2-tailed Student's *t-*test.

To determine whether adaptive immune responses are altered alongside the remodelling of the CD11b^+^ myeloid subsets, we next analysed T cell populations in the DRG and spleen at day 14 and day 28 following paclitaxel treatment. At day 14, flow cytometry analyses of T cell subsets in the DRG revealed no significant difference in CD4^+^ T cells (T helper) (Fig. 5A), however we observed a significant increase in CD8^+^ T cell (cytotoxic T cells) subsets although cell number was very low (Fig. 5B). Instead, at day 28, no changes were observed in T cell subsets compared with control DRG, indicating that early stages of paclitaxel-treatment might be associated with transient alterations in adaptive immune composition although minimal. Given the limited number of T cells detected in the DRG, we next assessed adaptive immune populations in the spleen to determine whether peripheral immune subsets exhibit more pronounced alterations. In contrast to the DRG, no changes were observed in splenic T cell subsets at day 14 (Fig. 5E–G). However, at day 28, paclitaxel treatment resulted in a reduction in regulatory T cells (Tregs) (Fig. 5J), while other T cell populations remained unchanged (Fig. 5H and I). This selective decrease in Tregs, in the absence of broader T cell alterations, suggests a specific loss of immunoregulatory control rather than a global alteration of adaptive immunity.

**Fig. 6:**
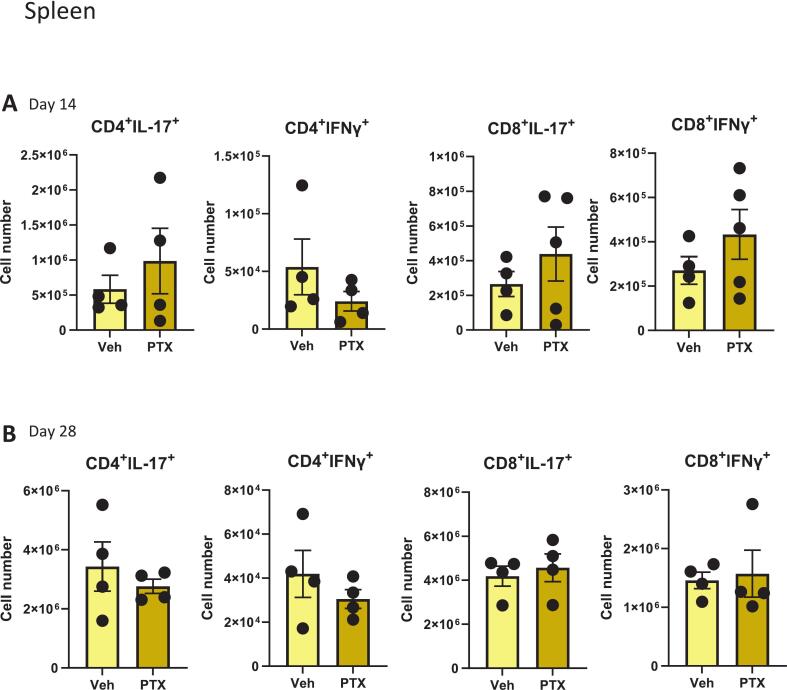
Paclitaxel treatment affects neither IL-17, nor IFNg production in splenic T cell subsets. A bar graphs represent splenic CD4^+^IL-17^+^, CD4^+^IFNγ^+^, CD8^+^IL-17^+^ and CD8^+^IFNγ^+^ at day 14 and B at day 28, n = 4–5. Data are presented as mean ± SEM.

In this context, CD8^+^ T cells are a known source of interferon-γ (IFNγ), which can promote macrophage activation, while reduced Tregs numbers may permit increased pro-inflammatory signalling, including pathways associated with interleukin-17 (IL-17). Therefore, we analysed IFNγ and IL-17 expression in CD8 but also in CD4 as they might contribute to the pro-inflammatory milieu. However, our flow cytometry analysis of both IL-17 and IFNγ in CD4 and CD8 revealed no changes after paclitaxel treatment compared to vehicle controls (Fig. 6A and B) This occurs in parallel with the emergence of Gal-3-expressing macrophages and remodelling of splenic CD11b^+^ myeloid compartment.

Together, these findings indicate that paclitaxel induces a delayed remodelling of adaptive immunity in peripheral compartments, characterised by reduced immunoregulation, which may contribute to the maintenance of neuroinflammation and persistent pain mechanisms in the DRG.

### 3.6 Paclitaxel does not affect adaptive immune responses in inguinal lymph node

**Fig. 7:**
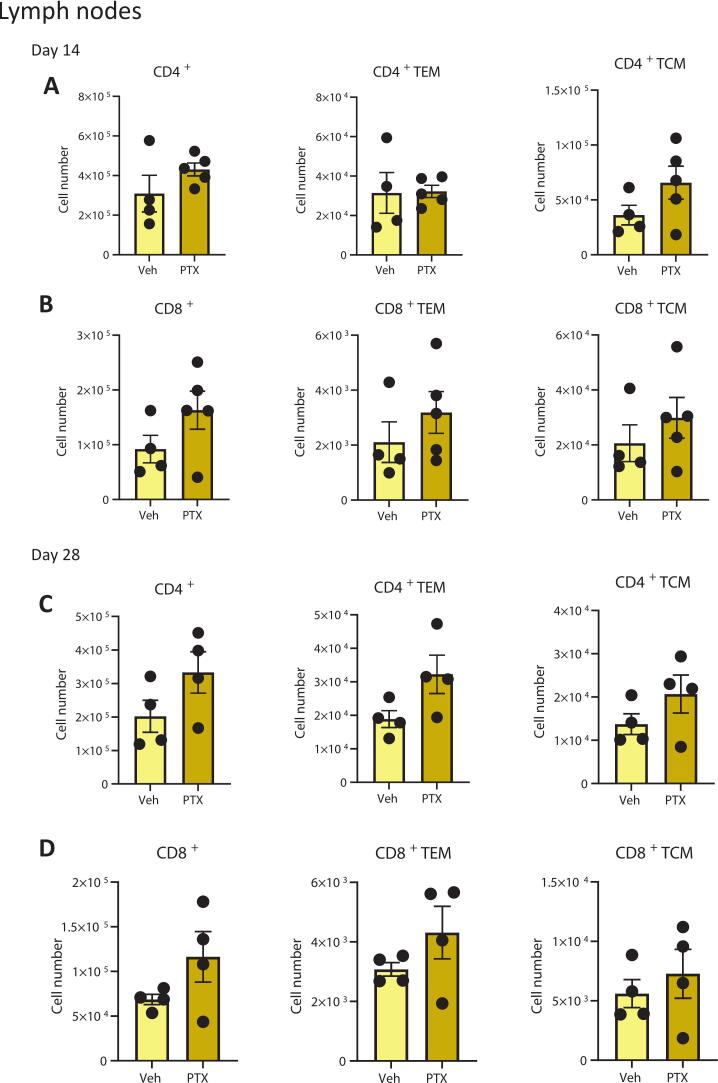
Paclitaxel treatment does not affect adaptive immune cells in inguinal lymph nodes. A bar graphs represent CD4^+^, CD4^+^ T effector memory (CD4+ TEM; CD4^+^CD62L^low^CD44^hi^), and CD4^+^ T central memory (CD4^+^ TCM; CD4^+^CD62L^hi^CD44^hi^) in inguinal lymph nodes at day 14 after paclitaxel treatment. B bar graphs represent CD8^+^, CD8^+^ T effector memory (CD8^+^ TEM; CD8^+^CD62L^low^CD44^hi^), and CD8^+^ T central memory (CD8^+^ TCM; CD4^+^CD62L^hi^CD44^hi^) in inguinal lymph nodes at day 14 after paclitaxel treatment, n4–5. C no changes in CD4^+^, CD4^+^ TEM nor in CD4^+^ TCM subsets at day 28. D No changes in CD8^+^, CD8^+^ TEM nor in CD8^+^ TCM subsets at day 28, after paclitaxel treatment, n = 4. Data are presented as mean ± SEM.

To determine whether paclitaxel-induced alterations in adaptive immunity extend to other secondary lymphoid organs, we analysed T cell populations in the inguinal lymph nodes at day 14 and day 28 following treatment. At both timepoints, flow cytometry analyses revealed no significant differences in T cell subsets between paclitaxel- and vehicle-treated groups (Fig. 7A–D). This included CD4^+^ and CD8^+^ T cells, as well as CD4^+^ T effector memory, CD4^+^ T central memory, CD8^+^ T effector memory and CD8^+^ T central memory, which remained comparable across conditions.

Together, these findings suggest that adaptive immune responses to paclitaxel are spatially organised**,** with early and transient changes occurring locally within the DRG and delayed alterations in immunoregulatory populations in the spleen, while lymph node compartments remain unaffected.

## 4 Discussion

This study shows that paclitaxel-induced mechanical hypersensitivity in male mice is associated with accumulation of macrophages in the DRG at 2 weeks from the last injection. Specifically, pro-inflammatory macrophage phenotype (MHCII^+^CD206^−^) prevails over MHCII^+^CD206^+^ macrophages, and Gal-3-expressing macrophages populate the DRG. The presence of Gal-3-expressing splenic monocytes and DRG macrophages at 2 weeks after termination of paclitaxel treatment suggests a pro-inflammatory, protective role for Gal-3. Indeed, the role of Gal-3 in-vivo depends on the type of damage and the context of organ damage (Hakim et al., 2025; Henderson and Sethi, 2009). Our data suggests that macrophages expressing Gal-3 exert a pronociceptive action through the release of Gal-3 which is a TLR-4 receptor ligand and can activate of TLR-4 receptor in either neurons or macrophages to facilitate nociceptive signalling. Such macrophages are supported by proinflammatory macrophages which are likely to maintain nociceptive neuron activity via releasing proinflammatory cytokines and prevail over macrophage-mediated mechanisms that alleviate CIPN neuropathic pain through the release of anti-inflammatory cytokines (Singh et al., 2022).

The onset and duration of paclitaxel-induced mechanical hypersensitivity that we observed match reported data in the mouse (Bacalhau et al., 2023). Although, the cumulative dose used in this study is higher than is some studies (Chen et al., 2011; Smith et al., 2004), a similar dosing regimen was used by others (Sankaranarayanan et al., 2023). Paclitaxel damages peripheral nerves and accumulates in the DRG. Proteomic analysis of rat DRGs isolated before the development of neuropathy and after fully developed neuropathy has revealed changes in protein associated with mitochondrial dysfunction (de Clauser et al., 2022). This data supports evidence of the presence of mitochondria with atypical, swollen morphology in preclinical models of CIPN at the level of the saphenous nerve, sciatic nerve, DRG, and sensory axons in the dorsal root (Trecarichi and Flatters, 2019). In neurons, paclitaxel enhances the sensitivity of TRPV1 receptor through activation of TLR-4 (Li et al., 2015) and increases the expression of sodium channels (Nav1.7 (human) and Nav1.8 (mouse) (Chang et al., 2018) and calcium channels and decreases expression of potassium channels (Li et al., 2017; Okubo et al., 2011; Zhang and Dougherty, 2014).

Furthermore, in the DRG, chemotherapeutics result in induction of proinflammatory cytokines in neurons and satellite cells (Kim et al., 2019) and activation of endothelial cells and promotion of monocyte/macrophage infiltration in DRG (Old et al., 2014). Indeed, paclitaxel induces increase in CCL2 expression in small nociceptive DRG neurons and CCL2 promotes infiltration of CCR2^+^ monocytes (Makker et al., 2017; Zhang et al., 2016). In this study, both splenic monocyte numbers and Gal-3-expressing monocytes were higher following paclitaxel treatment, though circulating monocyte numbers were not affected, suggesting that monocyte-derived macrophages may contribute at least in part to cell accumulation in the DRG.

Normally, DRG macrophage populations includes perivascular macrophages which are self-maintained and parenchymal macrophages which are constantly replaced by circulating monocytes (Lund et al., 2024). Perivascular macrophages play an important role in monitoring the blood-DRG barrier permeability and restricting access of circulating molecules into the DRG parenchyma (Lund et al., 2024). However, whether Gal-3-expressing macrophages identified in our study correspond to the vasculature-monitoring population remains to be explored.

Chemotherapeutics cause systemic immunosuppression, and paclitaxel increases circulating CD4+ and CD8+ T cell populations (Makker et al., 2017). We saw an early and transient increase in CD8^+^ T cells within the DRG, followed by a delayed reduction in splenic Tregs. Such DRG/spleen pattern supports a model in which adaptive immune responses are initially engaged locally, in DRGs, though T-cell numbers remain small. Then, at later stages, T cells feature systemic reduction in regulatory capacity. Interestingly, we detected no adaptive immune changes in the lymph nodes, which is likely due to lymph node's role in coordinating antigen-driven adaptive immune responses. Therefore, lack of detectable changes in lymph nodes suggests that paclitaxel-induced immune remodelling is driven by systemic inflammatory cues rather than by antigen-specific activation. This interpretation is consistent with the delayed expansion of splenic myeloid population and reduction in splenic Tregs.

Furthermore, although we detected no changes in IL-17 and IFNγ production in splenic T -cells, T cells reprogramming could have occurred locally within the DRG. Indeed, paclitaxel treatment promotes T cells recruitment in DRGs, alongside macrophage accumulation (Krukowski et al., 2016; Liu et al., 2014; Makker et al., 2017; Zhang et al., 2016). This compartment specific immune remodelling is consistent with our observation that adaptive immune cells differ among tissues such as DRG, spleen, blood and lymph nodes.

Whilst we provide insights into temporal and spatial organisation of immune cells in a model of CIPN, two limitations should be acknowledged. The first limitation is that immune profiling experiments were performed exclusively in male mice whilst immune mechanisms can be sex dimorphic. Indeed, macrophage and microglia- mediated mechanisms appear to predominate in males, whereas T cell dependant mechanisms play a prominent role in females (Mapplebeck et al., 2016; Sorge et al., 2015). The second one is that small sample size impacted on detection of subtle changes in immune profile and cytokine production in DRG. In follow up studies, we will evaluate possible differences in immune cell profiling in female mice and increase cell yield to expand cytokine characterisation. Nevertheless, we show a coordinated immune response after PTX treatment.

Overall, this study shows a time-dependant and compartmentalised immune responses characterised by the presence of pro-inflammatory macrophages in the DRG and a reduction in regulatory T cells in concomitance to fully developed allodynia following paclitaxel treatment. Together these results support the notion that neuroimmune interactions are critical for neuropathic pain maintenance.

## CRediT authorship contribution statement

**Lynda Zeboudj:** Writing – review & editing, Writing – original draft, Methodology, Formal analysis, Data curation, Conceptualization. **Marzia Malcangio:** Writing – review & editing, Writing – original draft, Supervision, Funding acquisition, Conceptualization.

## Authorship

**LZ** and **MM** conception and design of the study

**LZ** acquisition of data, or analysis and interpretation of data.

**MM** and **LZ** Drafting the article or revising it critically for important intellectual content.

**MM** and **LZ** Final approval of the version to be submitted.

## Declaration of competing interest

The authors declare that they have no known competing financial interests or personal relationships that could have appeared to influence the work reported in this paper.

## Data Availability

All data used in this manuscript are available upon request
